# Individual identities and stigma inequalities: insights from the experience of people affected by podoconiosis in Rwanda

**DOI:** 10.1186/s12939-025-02638-5

**Published:** 2025-10-07

**Authors:** Jean Paul Bikorimana, Josephine Mukabera, Gail Davey, Peter John Mugume, Papreen Nahar

**Affiliations:** 1 Centre for Gender Studies, College of Arts and Social Science, University of Rwanda, Kigali, Rwanda; 2 Centre for Global Health Research, Global Health and Infection Department, Brighton and Sussex Medical School, University of Sussex, Brighton, United Kingdom; 3 School of Public Health, Addis Ababa University, Addis Ababa, Ethiopia; 4 Centre for Conflict Management, College of Arts and Social Science, University of Rwanda, Kigali, Rwanda; 5 Centre of excellence in biodiversity and natural resources management, College of Science and Technology, University of Rwanda, Kigali, Rwanda

**Keywords:** Stigma, Individual identities, Health disparities, Gender, Socio-economic status, Podoconiosis, Rwanda

## Abstract

**Introduction:**

Podoconiosis is a Skin Neglected Tropical Disease (SNTD) that affects impoverished individuals in tropical regions. While there is a substantial understanding of the stigma associated with podoconiosis, little is known about the podoconiosis-related stigma experience based on individual identities, such as gender, class, age, location and physical ability. Due to the power differentials associated with these identities, individuals experience health problems differently, resulting in health disparities. This paper aims to discuss the inequalities related to podoconiosis stigma due to individual identities, informing policies and practices to reduce podoconiosis stigma-related disparities.

**Methods:**

This paper draws on a qualitative research approach to explore how individual identities shape the experience of podoconiosis stigma among affected people. Qualitative methods, including participant observation, interviews, focus group discussions, and key informant interviews with persons affected, family members, community health workers, and representatives, were employed. Data were thematically analysed.

**Results:**

Our findings reveal the complex nature of podoconiosis stigma-related inequality rooted in individual identities. We identified three main themes: (1) the importance of cultural norms and traditions in shaping social positioning, (2) Uneven stigma experience, and (3) the importance of one’s social positioning in coping with stigma due to podoconiosis. Certain stigmatised individuals or groups face higher levels of stigma than others. Affected individuals are often associated with culturally defined identities. Those with oppressive identities experience significantly more stigma compared to those with positive identities, because of cultural interpretations linked to class, ability, gender, and age. Poor married women and men, young girls, and disabled individuals with podoconiosis encounter greater stigma than their peers.

**Conclusion:**

This paper illuminates that the podoconiosis stigma inequalities are shaped by individuals’ identities related to gender, age, economic status, and bodily ability. Individuals with oppressive identities endure more stigma than others., and this differential stigma experience enhances the understanding of how disparities in stigma associated with podoconiosis or other SNTDs underpin health inequities. Such insights suggest integrating interventions to reduce podoconiosis stigma with others, such as gender equality education, economic empowerment programs, fostering positive identities and social inclusion, thus reducing disparities.

## Introduction

Podoconiosis is a gene-environmental disease that causes massive swelling of the feet and legs. It affects genetically susceptible individuals with prolonged exposure to irritant soil [1]. Podoconiosis affected vulnerable individuals with poor living conditions, mainly in tropical and sub-tropical regions. The evidence shows the presence of podoconiosis in 17 countries worldwide (12 in Africa, three in Latin America, and two in Asia) [2]. The national mapping for podoconiosis conducted in 2018 reported 7,000 cases of podoconiosis in Rwanda, and the cases were found in 30 districts of the country [3]. Rwanda is a low-income country located in East Central Africa with a total population of 13 million, and the majority of 62% are involved in farming activities [4]. 

Podoconiosis is one of the causes of lymphedema, and the World Health Organisation has incorporated it into the strategic framework for SNTDs, focusing on lymphedema management [5]. The interventions include a basic package of lymphedema management, such as hygiene, skin care, compression and shoe wearing [5]. These interventions are effective in managing podoconiosis and improving the quality of life for persons affected[6, 7], but are not available in many endemic countries.

Affected people face various adversities, including socio-economic hardships, psychological and emotional problems and social stigma [8]. Stigma experience due to podoconiosis is common among affected individuals and their relatives. Stigmatised individuals experience social exclusion, broken social interactions with others, and deprivation of their rights, which has a profound negative impact on their health and well-being [9–13]. Those individuals endure a range of adversities, such as being denied job opportunities, public insults, familial rejection and restricted social participation, affecting their overall well-being status. Also, they experience self-stigmatisation and exclude themselves from society [9]. Studies have reported experiences of affected people who dropped out of school, discontinued receiving healthcare services, or quit their jobs because of stigma[14, 15], creating landscapes for the inequalities, sustaining poverty, and hampering the well-being of affected families, leading to health disparities. These aspects of podoconiosis stigma —inequalities, poverty and poor well-being— are important to the interventions and strategies aiming to promote health equality and well-being, including global Sustainable Development Goals (SDGs), particularly SDG 1 [No poverty: End poverty in all its forms], SDG 3 [Good Health and Well-being], and SDG 10 [Reduced inequality] [5]. Therefore, it is essential to understand how podoconiosis stigma operates among affected people.

Podoconiosis stigma or stigma related to other skin NTDs at large is embedded in inequality and has the potential to exacerbate existing inequality because of individual characteristics and social structures. Across cultures, individual characteristics, including social identities and economic structures, shape people’s experiences [16, 17]. Social identities refer to the multiple factors that shape the ways people view themselves and how they are viewed by others in society [18]. The concept of social identities highlights how people are culturally grouped and belong to different social memberships and how individuals with certain identities are treated, viewed and considered in a given society [18, 19]. People are attached to multiple social identities, such as gender, age, economic status, ability, residency, and employment status. These identities are culturally constructed, shaping power relations and social hierarchy among community members. In many cultures, power differentials exist around patterns of gender, age, socioeconomic status, or geographical location, creating inequalities [20]. 

Those who carry minority identities experience certain levels of disadvantage and oppression. They have less decision-making power and the opportunity to unfold their potential, and are socially positioned in inferior positions [16]. 

Evidence shows the importance of social identities in shaping health experiences and contributing to health disparities [19]. Some social identities are linked with healthy living, whereas others experience disadvantages and oppression. Some individuals may fail to adopt healthy practices because of their social identities. For example, individuals with minority identities lack access to resources and the opportunity to afford basic services, including health services [21]. Those with marginalised identities experience poor living conditions, including a lack of access to health care services, clean water, and poor housing and nutrition[22, 23]. Consequently, those with stigmatised diseases may not have chances to improve stigmatising situations, resulting in health inequalities.

Furthermore, studies have documented a connection between individuals’ identities, including those related to gender, age, education, economic status and the experience of podoconiosis stigma [13, 15]. Some studies have reported those social categories as risk factors of stigma associated with podoconiosis and other skin NTDs [15]. For example, gender differences were connected with a high level of enacted stigma because of cultural constructs[11]. Podoconiosis stigmatised individuals with devalued identities are prone to experience greater stigma. However, the studies that focus on differences in podoconiosis stigma within affected individuals are scarce.

The focus of previous studies has been limited to stigmatising features and beliefs about podoconiosis, factors related to podoconiosis-related stigma, experiences and forms of podoconiosis stigma, and the intersectional experience of podoconiosis stigma [9–13, 15]. There is a tendency to homogenise stigmatisation across affected people. For example, causal beliefs and negative attitudes held by community members towards podoconiosis, disability, and bodily disfigurement have been linked to stigma [11]. Studies have also compared the experience of podoconiosis stigma between affected and non-affected individuals [24]. Affected people were found to experience a greater experience of stigma than non-affected people did. The scarcity of studies on the influence of social identities on the stigma associated with podoconiosis hinders our understanding of the nuances of inequalities stemming from this stigma. Consequently, it prevents the development of targeted interventions to alleviate podoconiosis stigma-related inequalities.

Our study aims to discuss how social structures and identities contribute to the inequality in experiencing podoconiosis among affected people, to inform policies and practices in relation to the reduction of Stigma associated with podoconiosis and other skin NTDs, contributing to the programme targets of the NTDs road map 2030 to reduce stigmatisation perpetuated towards affected people.

### Objectives of the study

The data used in this study were part of a large research project on exploring the lived experience of people affected by podoconiosis in Rwanda [25]. It had six main objectives; (1) to explore explanatory and cultural contructions of podoconiosis, (2) to understand health seeking behaviours of people affected by podocniosis, (3) to explore gender difference in the experience of podoconiosis suffering among affected people, 5) to study the specific dynamics and dimensions of Stigma experienced by affected people, and 6) to understand the social, emotional and economic consequences of podoconiosis on the life of affected people. The data reported in this paper are related only to the fifth objective, with a focus on two specific objectives: (1) to understand cultural constructs around individual identities, (2) to explore individual identity differences in experiencing podoconiosis stigma, to shed light on inequality embedded in podoconiosis stigma to provide suggestions for promoting the overall well-being of all affected individuals.

## Methods

### Design

This is a qualitative study, and a range of methods—participant observation (PO), in-depth interviews (IDIs), key informant interviews (KIIs), and focus group discussions (FGDs)—were used to obtain an in-depth understanding of how social identities shape podoconiosis stigma inequalities.

### Study setting and participants

This study was conducted in Nyamasheke district in the western province, which has the largest number of people affected by podoconiosis in Rwanda. Nyamasheke district is rural, and most people practice subsistence agriculture. The majority of people (53.3%) in the district are female, and patriarchal principles outweigh modern gender equality principles. The proportion of the population over 60 is relatively low (5.6%), whereas almost 62% of the population is under 25[4].

The study participants consisted of persons with podoconiosis (Table 1), family members, community representatives, healthcare workers, community health workers (CHWs) and traditional healers. CHWs are community members trained by the Ministry of Health to provide basic medical care and health education.

**Table 1:** Socioeconomic characteristics of individuals with podoconiosis

ID	Age	Gender	Marital status	Education (years)	Religion	Category of wealth	Duration of illness (years)
1	25	F	Unmarried	6	EMLR	1	6
2	52	F	Separated	0	Catholic	1	29
3	45	F	Married	0	Pentecost	2	21
4	43	F	Married	0	Catholic	3	21
5	48	M	Married	6	Catholic	2	38
6	70	M	Married	6	Catholic	2	20
7	48	F	Married	0	Catholic	3	13
8	45	F	Married	0	Catholic	1	30
9	66	F	Widow	4	Catholic	2	39
10	61	F	Widow	0	Catholic	1	44
11	68	M	Married	0	Catholic	1	40
12	60	F	Separated	0	Catholic	1	37
13	25	F	Unmarried	5	Catholic	1	10
14	37	F	Separated	1	Catholic	1	1
15	80	M	Married	9	Catholic	1	1
16	55	M	Unmarried	1	Catholic	1	37

Categories of Wealth ranged from 1 = poorest, 2 = poor, 3 = rich, 4 = richest

*EMLR* Église méthodiste libre au Rwanda, *F* Female, *M* Male

The participants were purposively recruited, and the principle of maximum variation was ensured by including participants with various characteristics, including gender, age, duration of illness, education level, religion, and wealth categories [26]. The recruitment process was performed in phases, and one participant was selected at a time for the interview and participant observation. Key informants were selected to participate in FGDs and KIIs. Details on the recruitment process have been provided elsewhere [13]. The data collection was guided by the principle of saturation, and the interviews and FGDs were concluded when there was no addition of new data [27]. However, the data collection was limited by the short duration of participant observation. Because of the nature of the study, the clinical diagnosis of podoconiosis was conducted after data collection with persons affected for confirmation [28]. 

### Data collection

This study was part of a large social science research project conducted under the Social Science for Severely Stigmatising Skin Diseases (5 S) Foundation [29]. The research fieldwork ran from the 31 st March to the 23rd December 2022. The research team consisted of researchers with academic and research backgrounds, and they were JPB supervisors for his PhD. JPB was trained in qualitative research methodology, and he conducted the data collection and analysis.

The data collection happened in parallel with analysis, and many methods as indicated in the methods section were used to collect data. IDIs were conducted for sixteen persons affected and four family members to explore the stigma experience and how affected people make sense of the everyday experience. All participants preferred to have interviews in their homes, and the schedule for interviews was arranged beforehand. Subsequent interviews were conducted for clarity and further exploration when needed [27]. We used semi-structured interviews and an interview guide was developed with the help of experts and contains topics related to stigma.

Seven persons affected were purposively recruited [considering those living in easy-to-reach locality] to participate in the observation from those who previously participated in the in-depth interviews for triangulation purposes. All participants consented to participate, and each time permission was sought for observation. An interval of one week was set for the next observation sessions, and the observation schedule for observation time was set by the participants accordingly to their availability [30]. Each person was visited three times on different days (in the morning, midday, and evening) to capture various aspects of their daily lives. At home, JPB and a field guide were engaged in natural conversations with family members and helped with the activities happening at the moment, making the visit natural. However, we realised that the presence of JPB and the field guide during the observations altered the situation at home. A session was conducted to explain to persons affected and family members that they should continue their activities. A checklist was used for PO; however, observations were made beyond the checklist. Various aspects were observed, including main and rare activities at home, interactions, roles, home environment, behaviours and practices, and field notes were taken, mainly after the observation.

KIs and FGDs were conducted with six key informants and 32 CHWs, respectively, to understand the sociocultural context of the experience of stigma and categories of people. KI and FGDs were conducted after interviews with persons affected and their family members to gain a clear understanding of cultural and structural contexts and ways in which they shape the stigma experience of persons with podoconiosis. The narratives of persons affected informed the recruitment of informants, and KIs were conducted in different sites, including offices and homes, based on their availability. Regarding FGDs, CHWs were invited, after they had signed the consent form, to participate in the discussion in the conference rooms provided by the health centre in the area, and we had one FGD per day, and notes were taken by a research assistant. Techniques, such as probes and prompts, that encouraged study participants to generate in-depth data were employed [31, 32]. 

The interviews and FGDs were conducted in a local language, Kinyarwanda, and audiotaped using an audio recorder. Data from each participant category and different sources were triangulated to understand persons affected’s experiences with stigma [33]. Data were stored confidentially and accessed by only authorised persons.

### Data analysis

Audio data were transcribed into text-based data and analysed thematically [34]. JPB and research assistants transcribed the audio data. Before the transcripts were imported into NVivo version 20, JPB played the recordings several times while the transcripts were read to cross-check them for validation. The transcripts were kept in the language of the interviews, Kinyarwanda, but the coding process was performed in English.

The codebook was computed and discussed among the research team for input. Quotes were translated into English, and bilingual [Kinyarwanda and English] experts were consulted during translation to preserve the original meanings of the data.

All the data sources—field notes, audio and transcripts—were triangulated by checking for similarities and differences [33, 35]. The data were coded both deductively and inductively. While deductive coding helped ensure that the data were aligned with the study’s objectives, inductive methods helped generate themes grounded in the participants’ perspectives [36, 37]. Codes were organised and categorised under themes. The main themes were described and checked for relevance to the stigma experience of affected people before writing [34]. 

### Trustworthy of the study

Interpretations of the key themes were cross-checked among participants, and revisions were made accordingly [35]. JPB was a Rwandan but new to Karengera sector, Nyamasheke district, and had socioeconomic and educational background differences from the study participants. He spent the first month living in the community to explore and familiarise himself with the community members, contributing to enriching the data [30]. 

## Results

### Participants' profile

Sixteen women and men with podoconiosis participated in our study (Table 1). Their ages ranged from 25 to 90 years old, and a large number of them (*n* = 13) had podoconiosis for more than ten years. Most of them were female (*n* = 11), and the majority were still doing farming activities (*n* = 15). Forty-three non-affected people, relatives (*n* = 5), community representatives (*n* = 6) and CHWs (*n* = 32) participated.

The data analysis revealed three main themes: (1) The importance of cultural norms and traditions in shaping social positioning. (2) Uneven Stigma Experience due to cultural perspectives attached to individual identities in understanding disparities in the stigma experience associated with podoconiosis, (3) The importance of one’s social position in coping with stigma due to podoconiosis, representing the experience of podoconiosis stigma due to individual identities endured by persons affected.

#### Theme 1. Cultural norms and traditions of individual identities and social positioning

In our study, participants reported many cultural constructs surrounding individuals’ identities and gender, age, ability and economic-related constructs were commonly said to shape the levels of hierarchy among individuals, and the experience of stigma. In Nyamasheke, people believe in and are firmly attached to their cultural traditions, values and norms. It was clear how those cultural values and norms dictated perceptions held by community members towards categories of individuals in everyday life. Regarding gender, participants discussed many constructs that contribute to how men and women are affected by podoconiosis differently, and social position, religious reasons and bodily/symbolic representation were dominant.

Participants discussed how social position influences the differences between men and women, with men typically holding higher positions than women, and they employed a brain/heart metaphor to illustrate these differences. The meaning of the brain/heart metaphor was grounded in the culturally constructed division of labour between men and women. Being a man was commonly portrayed as the ‘brain’—positioning men as controllers and regulators of everything in their families. These positions gave men supremacy over women, justifying the respect of men. On the other hand, being a woman was portrayed as the ‘heart’, making women the engines that ran domestic chores and simultaneously served as caregivers, helpers, and nurturers. Participants shared how such a construction of a man as a brain places women in a lower position in society.

*“A man is like a king here; he is respected*,* and even women are aware of that; no woman would dare say she is a family chief. They say that a man is a chief of the family.” (CHW*,* M*,* 62 years old)*.

*“A woman is the heart of a household; this means that she needs to take care of everything at home and do every domestic chore. It is the same as blood circulation in all body parts.” (CHW*,* F*,* 61 years old)*.

Another way to explain the difference between men and women arose from religious beliefs. The majority of the community members are Christians, and religious faith played a role in shaping men’s supremacy and women’s inferiority. It was commonly believed in the community that because a man was the first creature, according to the bible, this put him in a superior position, justifying the suppression of women. Participants explained how religious beliefs around creation were used as a tool to confirm the dominance of men over women.

*“Men and women are different because of how God created a man and woman…God created a man first and then a woman to help him*,* so there is a reason for this order. A man needs to be respected.” (CHW*,* F*,* 37 years old)*.

Symbolic representation was another way in which men and women were distinguished, rooted in the differences between their bodies. Beauty was one of the traits used to assess women’s social acceptance, but not men’s. Some participants discussed how men had specific criteria for choosing a woman for marriage, with bodily beauty being among them; those whose beauty was disfigured by podoconiosis faced a significant burden, including stigmatisation.

*“It looks awful for women to have swollen legs. All people would be concerned about her*,* and it is not like for men.” (person affected*,* F*,* 60 years old)*.

Also, the participants explained how the terms ‘man’ and ‘woman’ were used to describe performance in society, not merely to refer to an individual but also to one’s achievement or failure.

They noted that the term “man” was widely used to refer to a male or female with outstanding achievements, which was not the case for the term “woman”.

*“Also*,* women or young boys can be called men because of their physical strength or performance*,* which is considered important. The word ' man is used to talk about someone with great achievements.” (CHW*,* M*,* 46 years old)*.

The presence of a man in a household was connected with the dignity and protection of the household. The participants explained that households without men, whether a husband or a son, experienced instances of disrespect and were portrayed as incomplete. Also, some widowers or those separated from their husbands expressed feelings of being humiliated because they did not have husbands.

*“When someone becomes a widow*,* she starts experiencing a kind of loss of dignity*,* and people who used not to dare to come to that household start coming because there is no man in that house. It is a kind of disrespect.” (CHW*,* F*,* 56 years old)*.

*“People do not treat you well if they know you are a widow. I used to feel respected when my husband was still alive*,* but now I feel a big gap in my life because of his absence.” (Person affected*,* F*,* 60 years old)*.

Contradictory views on the suppression of women were observed, however. Some participants believed that the government policy of gender equality had altered their perspectives on the capabilities of women, for example, that women had become involved in physically demanding work or providing for households.

*“Women in this area have started doing men’s activities; as we can see*,* some women are now the providers of their families*,* whereas their husbands do nothing. We know women who work hard*,* doing vegetable businesses and arts to earn money. Although they earn little money*,* they do many things with it in their households.” (KI*,* M*,* 60 years old)*.

However, the participants reported that many women still positioned themselves as subordinates to their husbands because of trans-generational training from grandmothers. One woman pointed out how grandmothers used to educate their granddaughters to respect their husbands:

*“Our parents used to tell us that men are to be respected because they are few. In addition*,* we grew up with that conviction and stuck with it even in our marriage.” (CHW*,* F*,* 56 years old)*.

Gender-related identities set landscapes for privileges and disadvantages across individuals, leading to inequalities among women and men. Perceptions attached to being a man or a woman contributed to occupying certain social positions. This shaped the everyday experience of individuals in terms of how they viewed themselves or were viewed by others, affecting the ways in which affected individuals experienced podoconiosis stigma.

The participants also reported cultural constructs related to age that shape individuals ' daily experience of podoconiosis stigma, including prospects for those of a younger age, respect and care for those of an advanced age. They explained that people were culturally required to respect and care for their elders [> 60 years], and stressed that children and grandchildren were responsible for caring for their parents and grandparents. Also, the elderly were not expected to work in the field; they were judged based on their past lives, considering them as people who had finished living and were waiting for death. On the other hand, young individuals [< 18 years old], middle-aged individuals [between 19 and 45 years], and adults [> 45 years but < 60 years] were judged prospectively, socially and economically. The participants discussed how future prospects were a concern for those of a young age.

*“Compared with elderly individuals*,* an enormous burden is placed on the young population*,* as elderly individuals have lived their lives and have children. It is a worrying situation for youth*,* particularly girls*,* as they are concerned about the future and how they will find husbands.” (Person affected*,* M*,* 52 years old).*

On the other hand, participants talked about current challenges in caring for elders. Because of modernisation, in certain low-income families, older adults have started being seen as a burden because they are no longer physically productive. This was described as cultural wrongdoing that needed to be avoided.

*“Elderly individuals may wish to pass away*,* as they believe they will become a burden due to their old age and inability to work.” (KI*,* F*,* 49 years old).*

Cultural norms related to age play a role in shaping one’s social position. Cultural methods contribute to determining the social position one occupies in a society and how one experiences podoconiosis stigma.

Regarding economically related cultural constructs, many constructs that shape the experience of podoconiosis stigma were reported, including constructs related to being poor or rich. The participants metaphorically explained the difference between better-off and poorer individuals using the relationship between a master and a slave. In Nyamasheke, economic status played a role in determining social status. Well-off individuals were treated with respect and considered life providers. On the other hand, poor people were described as slaves, linked with disrespectful treatment, and as people without constructive or positive thoughts. Participants discussed how the poor felt ignored because they are connected with negative attitudes such as laziness and poor mindsets, and those who perceived themselves as poor positioned themselves or were positioned by others in an inferior position.

*“Those who are considered poor in this area are slaves of those who are rich*,* because they use all their strengths to work for rich people” (KI*,* M*,* 60 years old)*.

*“The poor are ignored in this area and have miserable living conditions. They feel ashamed*,* and they refuse to interact with others in society; they isolate themselves from others” (KI*,* F*,* 40 years old)*.

Most participants shared that the difference between being poor and being well-off was clear. Constructs associated with economic status contributed to the experience of podoconiosis and well-being. While being rich afforded a high social status, there was neglect of being poor. Constructs related to physical ability were also commonly reported by the participants to shape the experience of podoconiosis stigma. The bodily ability to work was described as a requirement to live in this area and reflected the contextual living conditions, as it is a rural and mountainous region. On the other hand, those without employment or the ability to work were perceived as a burden. Tendencies such as begging, laziness and theft were ascribed to those who did not have things to do. Also, the ability to work has a gender dimension in the area, especially around marriage. As a pro-family community, marriage was culturally significant in the area. In addition to bodily beauty, working ability was also a requirement for marriage. Among the mainly uneducated men, a fiancée was selected based on the girls’ economic capacity and working ability. Men asked more than one girl to reveal their assets, including bank accounts, cash, cows, pigs or pieces of land.

*“In contrast to individuals considered incapable*,* individuals capable of working were treated with dignity. Generally*,* a person who does not work is always problematic to society.” (CHW*,* F*,* 43 years old)*.

*“As women*,* we need to work hard to be able to take care of our families*,* children*,* and husbands. Women’s money goes directly to domestic chores*,* and sometimes*,* men spend their money in bars drinking beer with others.” (CHW*,* F*,* 45 years old)*.

The meanings attributed to identities related to ability were crucial determinants of social status and were shaped by cultural values and contextual factors. The people in the region were dynamic and relied on subsistence agriculture in the high mountains and valleys. Being disabled in this context led to social prejudice and rejection for both impoverished men and women facing difficulties in work.

Social hierarchy prevails among individuals, and social identities contribute to the exacerbation of health inequalities among them. Those with marginalised identities experience greater social prejudices than others. Individual identities are culturally constructed and imbued with distinct meanings and interpretations for different people. Those with culturally devalued identities possess a low social status, rendering them susceptible to increased stigma due to podoconiosis.

#### Theme 2: Uneven stigma experience: Disparities in experiencing podoconiosis stigma because of individual identities

For the experience of podoconiosis stigma, the data related to this second theme of disparities in experiencing podoconiosis showed that affected people experienced a variety of stigma experiences, including felt and enacted, and the differences in the amount of their experiences. Participants asserted that the amount of podoconiosis experienced was connected with identities related to gender, economic status, age and physical ability. They talked about how some affected people experienced a greater amount of podoconiosis stigma than others because they were attached to demeaning and stigmatising identities, including being female, poor, young age and bodily incapacitated, as presented in the first theme of cultural constructs surrounding individual identities.

##### Gendered podoconiosis stigma

For the disparities in experiencing podoconiosis stigma, gender related social constructs were commonly mentioned to shape the difference in the amount of stigma experienced. The cultural expectations and structures surrounding gender, presented in first theme, influenced the extent and nature of stigma associated with podoconiosis, with women being more stigmatised than men. Participants discussed how affected women endured a greater number of insults, social exclusion, feelings of shame, and public ridicule due to swollen feet and legs. They explained that bodily disfigurement and disability from podoconiosis were associated with social prejudice against affected women. Concerns over bodily beauty were significant for women, given the cultural expectations for social acceptance. Many affected women noted that due to the visibility of symptoms, people feared physical contact and tended to gossip about them, further exacerbating feelings of shame. Both affected and unaffected participants reported that men faced fewer public insults. They explained that men were expected to be physically strong, which led to a belief that they would confront those who stigmatised them. One participant shared how she was exposed to podoconiosis stigma because of her gender.

*“Even in the community*,* people stopped to stare at me and talked about me because they thought I could not do anything about them as a girl. However*,* they could not talk about a man in the same way; in addition to men being like kings in this area*,* they also have the strength to fight whoever talks about them. For me*,* I accept it and let them talk about what they want” (Person affected*,* F*,* 25 years old)*.

Also, participants talked about how affected women experienced stigma related to marriage issues. They explained how affected women were subjected to separation from their husbands because of swelling of the feet and legs, and how women faced difficulties in finding partners. Participants reported that some husbands refused to be seen with their wives and started avoiding walking with them because of the swelling of their feet and legs. Husbands preferred to walk alone when they planned to meet new friends or attend important events. One woman shared how she hid her swollen legs and feet from her fiancé, fearing that he could break up with her.

*“I could not disclose to him (her fiancé) about my illness until we got married. I hid my illness from him by putting on shoes. After all*,* I had shoes that fit my feet at that time because I knew he would not marry me when he knew that I had legs like these”. (Person affected*,* F*,* 45 years old.)*

*“My husband refuses to walk with me to visit friends; he goes alone because he does not feel comfortable walking with a woman who has swollen legs.” (Person affected*,* F*,* 48 years old)*.

Cultural norms and values related to gender established power dynamics between affected men and women, rendering women more susceptible to stigmatisation. Men were culturally perceived as strong and faced less stigma compared to women, who were positioned lower in the societal hierarchy, thereby increasing their vulnerability to stigmatisation.

##### Economic status: intensified stigma of being poor

Participants reported that economic status affected their experience of podoconiosis stigma, and that poor people were more stigmatised than others. Participants stated that the extent of podoconiosis stigma varied between affected poor people and well-off people. They explained that among affected people, those who were poor were socially excluded and discriminated against by other people and that they isolated themselves because of low self-esteem. An increase in stigma was attributed to the stereotypes and living conditions of poor people.

The participants talked about how poor people were considered to have poor mindsets and blamed themselves for being responsible for inadequate hygiene.

*“People think we are not like them and do not know how to wash our bodies. In this village*,* some people talk about me*,* saying that the woman does not wash her legs properly*,* but I do not tell them anything.” (Person affected*,* F*,* 60 years old)*.

*“Sometimes I need to raise my hand to ask a question in a meeting*,* but I fear doing so. After all*,* I think that people could not hear me because I am poor and I have swollen legs*,* and this makes you feel that you are nothing.” (Person affected*,* F*,* 43 years old)*.

Some participants asserted that affected individuals live in poor conditions, making practising good hygiene difficult. They explained that podoconiosis causes wounds, cracks, and rough skin, which require thorough skin hygiene—something that poor Persons affected cannot afford due to their home environment. Based on observation, most poor Persons affected resided in small, dilapidated houses made of timber and mud, had earth floors, and slept on beds made of grass or traditional mats. Participants noted that these conditions hindered poor Persons affected from maintaining adequate hygiene. Furthermore, they could not afford sanitary materials, worsening their situation by causing offensive foot odour, which fuels stigma. Many participants reported that those with unpleasant-smelling feet faced greater social prejudice, shunning, and isolation than other Persons affected.

*“When the disease becomes severe*,* it is difficult to practice hygiene; Persons affected feel pain*,* develop wounds*,* and they cannot afford to buy shoes; these are all problems*,* and that is why they fear attending social events because their feet produce an unpleasant odour due to poor hygiene.” (CHW*,* M*,* 48 years old)*.

Economic-related identities intersect with other identities, [we discussed podoconiosis stigma experience and intersectionality elsewhere][13], creating disparities in the podoconiosis experience between poor and affluent Persons affected. Being poor was connected with stigmatising social perceptions, fuelling stigma for those who viewed themselves and were viewed by others as poor to bear a greater degree of podoconiosis than Persons affected who were considered well off.

##### Age-related stigma

Participants asserted that age-related identities also contributed to the differential experience of podoconiosis stigma among age categories, with individuals of different ages encountering stigma in varying ways. Participants noted that younger and middle-aged people faced more stigma than elderly individuals. Both affected and non-affected participants discussed how young or middle-aged Persons affected, particularly females, grappled with feelings of shame and social rejection. Young female Persons affected recounted their experiences of avoiding public places due to the fear of being seen with swollen feet at a young age. Additionally, affected women shared their experiences of feeling ashamed of their swollen feet and legs when they were younger, although these feelings lessened as they grew older.

*“I used to feel ashamed when I was still young*,* but now I have grown up*,* and it has become the usual thing that I have to live with; it is no longer a problem. Now*,* I cannot fear joining other groups of people. Yes*,* some people stopped and stared at me*,* but I ignored them and continued my journey.” (Person affected*,* F*,* 61 years old).*

Age-related identities played a role in shaping the experience of podoconiosis stigma and contributed to disparities in the stigma surrounding the condition. Being young or middle-aged with podoconiosis was linked to an inability to meet specific cultural expectations, such as ideals of bodily beauty, strength, or prospects, which in turn fostered social prejudices.

##### Ability: the intensified stigma of failing to fulfil one’s roles

The disparities in experiencing podoconiosis stigma due to physical ability included stigma related to diminished competency and productivity. Participants linked incompetence and poor productivity with difficulties in working and walking, fuelling stigma for those incapacitated because of podoconiosis. This ability-related stigma was connected with people’s daily lives and the geographical structure of the Nyamasheke district. The district is mountainous, and people live off farming. Healthy feet and legs were described as a requirement to live in this area, allowing them to climb up and down hills. Participants described how podoconiosis affected walking and working ability, and those who were severely incapacitated felt unworthy or useless more than others because it was difficult for them to fulfil their roles. They explained that those with severe swelling of the feet and legs were denied jobs or opportunities to participate in community work because they were perceived to be incompetent.

*“Because of my feet and legs*,* nobody in this area could offer me a job; they observe my feet and say there are no more jobs. So as a man*,* you feel that you are nothing*,* an unworthy man*,* because you cannot provide for your family.” (Person affected*,* M*,* 48 years old)*.

*“Nobody offers me a job when I ask for it. I remember when I went to search for a job*,* my husband was sick at that time*,* and we had nothing in our house. I looked for a job*,* but nobody allowed me to work…they looked at my feet and thought that I was not strong enough to cultivate*,* told me that I was sick*,* and I had to go for treatment first.” (Person affected*,* F*,* 45 years old)*.

Also, the loss of ability for affected women was among the reasons for separation from their husbands, marriage instability for women and difficulty in obtaining a partner for affected young girls. Some affected women talked about how their husbands stigmatised them because they were no longer able to work in fields or take care of domestic chores.

“*My husband would feel ashamed to walk with a person like me. He did not want me anymore; I was useless when my condition got bad; I would spend many days at home doing nothing. Then*,* he gave me a red* licence and told me to *“go and live with my mother because I cannot live with a woman who cannot do anything*,* while there are healthy women out there. There was no choice since I was jobless*,* I was not a teacher*,* I had no education*,* no money.” (Person affected*,* F*,* 60 years old.)*

Interestingly, some of the women who experienced marriage insecurity because of loss of ability confirmed their situation by blaming themselves for being useless to their husbands and families since they could not work in the fields nor take care of their children like other women, fuelling stigmatisation for them.

*“As a woman*,* you feel useless when you cannot take care of your family*,* children and husband*,* and men cannot accept living with a wife of that kind*,* who will not work in fields and bring anything home.” (Person affected*,* F*,* 68 years old)*.

However, one affected man talked about how his marriage was fractured because of his lost working ability and described himself as an unworthy man because of the loss of working ability due to podoconiosis. He had a conflict with his wife and was forced to move to a separate small house alone. He hardly had meals because he was not working to provide for his family.

*“My wife once told me that I am like a tree stump and refused to give me meals because I do not earn anything unless I can work and earn money to provide for my family; you cannot feel like a man.” (Person affected*,* M*,* 58 years old).*

Ability-related identities intersect with various identities, such as gender, poverty, and contextual factors, creating differences between incapacitated and capable Persons affected. Culturally, individuals were assigned different roles, and failing to fulfil these roles was deemed deviant. People blamed themselves for such deviations, and the feeling of failure heightened the stigma. Participants’experiences and views illuminate how cultural and structural constructs attached to individual identities, as they explained under the first theme, shape the disparities in stigma experience across persons affected, leading to unequal health outcomes.

#### Theme 3: Individual positioning and coping strategies

Individual identities related to gender, age, and economic status commonly affected the strategies used by affected people in coping with podoconiosis stigma, including Self-acceptance (normalisation and vulnerability), hiding illness and perseverance [*Kwihambira*]. The ability to employ a desired strategy varied among individuals, with some lacking the opportunity to use it.

Self-acceptance was commonly used to cope with podoconiosis among poor individuals. This strategy was based on social expectations and the ability to afford treatment to remove stigmatising features, intensifying stigma. Most participants said that they had learnt to accept their situation because they did not have the resources to seek treatment. They reported that poor Persons affected lacked the opportunity to use therapies. Therefore, they accepted their situation.

Poor Persons affected with severe disease normalised their situation because of the visibility of their symptoms. Normalisation and acceptance of their situation were solutions for them since they could not remove visible stigmatising features. There was a sense of vulnerability in the acceptance of the situation, which was rooted in the fact that their illness was visible, and they lacked the resources to seek effective therapies.

*“You know I cannot take away this illness from me; I cannot cut off my legs; for me*,* it is to accept it*,* and those who talk about me*,* they talk about the things they can see; it has become normal*,* I let them talk whatever they want.” (Person affected*,* F*,* 45 years old)*.

Individual identities linked to economic class and the severity of podoconiosis affected self-acceptance. Underprivileged Persons affected came to terms with their situation due to the absence of opportunities to address stigmatising traits, resulting in health disparities between impoverished and affluent Persons affected.

Hiding the illness.

Affected people reported the use of hiding the illness strategies to cope with podoconiosis, which was done in different ways, including covering feet and legs with clothes or footwear and avoiding going to public places such as marketplaces, neighbours, and schools to reduce stigma. They talked about how some individuals failed to hide their affected feet and legs, exposing them to stigmatisation. They explained that poor, affected women or men failed to hide their feet and legs because they could not afford to buy boots, closed shoes, appropriate long robes, fabrics or trousers. More specifically, affected women and men with severe swelling struggled to cover their swollen legs, increasing stigma. On the other hand, those from well-off families reported customising their footwear or clothes to cover affected parts, reducing stigma.

*“They do not laugh at me; where do they see me? People do not see my legs*,* and very few people are aware of my sickness because I do not share my problems with anyone. In addition*,* I always put on my boots*,* and they see me wearing them*,* and they do not know I have this illness.” (Person affected*,* F*,* 48 years old)*.

*“People laugh at us. Sometimes*,* I wear long fabric to cover my feet so that they (people) would not see them*,* but it is still observable. Because it is hard to hide my feet and legs. Others wear shoes*,* but for me*,* there are no shoes that can fit my feet like these.” (Person affected*,* F*,* 61 years old*).

Two women reported that they had never tried to hide their legs, and they let people talk about whatever they wanted because they knew the body could change at any time and that everyone could be affected.

*“I cannot hide my illness because it is not my fault to have this illness; it is my body that disappointed me.” (Person affected*,* M*,* 68 years old)*.

Hiding podoconiosis was thought to lessen stigma, yet those affected with marginalised identities struggled to utilise the hiding strategies. This approach proved more challenging for poor women with severe swelling compared to others, resulting in a heightened experience of stigma. This strategy was not within the means of poor women with severe swelling than for those in other categories, leading to a more significant experience of stigma.

Perseverance.

Participants reported the use of a perseverance strategy to cope with podoconiosis, and the aim of this approach was to eliminate stigmatising constructs by pushing themselves to work hard despite their illness to improve stigmatising constructs, such as being perceived as a lazy person or thief. Some participants talked about how they had to work in the fields regardless of their pain and swelling. Based on the participant observation, perseverance was motivated by the fact that working in the fields was one of the core social values in the area, and laziness was associated with social prejudices and judgment. Participants explained that some categories that affected people experienced more difficulties applying this strategy. Poor affected individuals worked mainly in fields that were far from their homes and reported challenges related to walking long distances and working. They talked about how they developed acute attacks [painful, inflammatory body responses] after spending days working in the fields. This led them to spend more days off at home. Similarly, affected people of advanced age are limited in using this strategy because of diminished physical strength. However, well-off individuals hire labourers so that they can perform light tasks, diminishing the risks of acute attacks and fatigue.

*“I feel tired while working in the fields*,* and my legs became too heavy to lift and hurt so much*,* but should I stop working? No*,* I have to push it to earn a living.” (Person affected*,* F*,* 60 years old)*.

Many affected people wanted to work hard to change their situation, but diverse individual identities worked together to limit their performance, trapping them in a cycle of problems. Being poor, having advanced podoconiosis and being older hampered the chances of adopting the strategy of perseverance to minimise stigma.

## Discussion

### Summary of findings

Individuals affected by podoconiosis who participated in our study experienced an unequal amount of stigma because of individual identities, and those with demeaning identities face greater stigma than those with positive identities, suggesting the disparities and inequalities embedded in podoconiosis-related stigma. Their stigma experiences included felt and enacted. Certain individual identities were associated with stigmatising cultural constructs, such as being perceived as a second creature, helper, carer, giver and nurturer attached to female gender; poor mindsets and thoughtful less attached to being poor, and incompetency, less productive, theft, and laziness attached to diminished physical ability and unemployment, fuelling the experience of podoconiosis stigma for vulnerable affected groups. Interestingly, certain women confirm their subordination and suppression as a cultural obligation of women. Also, our findings shed light on how individual identities shaped the strategies that affected individuals to cope with podoconiosis stigma. Affected people with poor resources failed to use strategies of their choice, exposing them to more stigmatisation compared to others, leading to health disparities.

### Cultural constructs of individual identities and podoconiosis stigma experience

Consistent with the existing literature about the connection of social identities and health-related stigma[38, 39], our findings demonstrated the connection between the stigma experienced by people affected by podoconiosis and their social identities. Some affected people are assigned oppressive identities, increasing the amount of stigma they experience. Individual identities are culturally constructed, and certain identities were associated with demeaning constructs, including those related to poverty, gender, age, and disability, fuelling social prejudice, judgment, exclusion and discrimination. These findings reflect the work of Weiss on stigma related to NTDs, underscoring that people affected by NTDs possess other stigmatising identities, shaping the stigmatisation and oppression they face [40]. For example, in Nyamasheke, such constructs are embedded in cultural perspective, religious faith, traditions, and normative grounds, and create hierarchies and power dynamics among individuals; those with minority identities suffer oppression, stigma, and discrimination. Our study provides a comparison with previous studies that reported the podoconiosis constructs associated with podoconiosis, including hereditary beliefs, loss of productivity, bodily disfigurement, and advanced stages of podoconiosis associated with stigmatisation [11]. The constructs in these studies have not necessarily reflected on individual identities, and it may seem that anyone exhibiting these characteristics would be stigmatised in a similar manner. Contrarily, we found that these stigmatising constructs are culturally constructed and interpreted differently depending on individuals’ identities and positions within the community. The difference between being rich and poor was explained using the historical relationship between a master and a slave, putting those perceived as poor in a low position as slaves, exposing them to the experience of stigma. While those perceived as rich received less stigmatisation, poor affected individuals were ignored and experienced low self-esteem, limiting their potential for societal participation. On the other hand, religious beliefs, patriarchal beliefs and gender expectations lay the foundation for the differences between women and men.

In most cases, symbolic representations and traditions attributed to women place them in a subordinate position, exposing those with podoconiosis to greater stigma than men, leading to health disparities across affected individuals.

The unpleasant smell and appearance associated with advanced disease seem to be common among many affected individuals. However, the stigma experienced by a poor adult mother with podoconiosis may be greater than that faced by a man with the same condition or by a well-off mother at the same stage of illness. The stereotypes linked to having smelly feet vary from those of affected individuals with different identities, including an elderly mother, a wealthy mother, a married woman, or a poor man. These findings reflect a vast body of literature demonstrating how cultural values, norms, and traditions shape self-identification and create landscapes of inequality and oppression [41]. In many parts of the world, poor individuals and women are relegated and experience humiliation. Women are bound by values and beliefs that are primarily rooted in cultural expectations and roles, such as caregiving, nurturing, and domestic responsibilities, which place them in inferior positions [42]. Women are often perceived as physically weak, assigned roles that are less demanding and sometimes associated with lower wages or going unrecognised. Podoconiosis exacerbates the oppression faced by poor women more than other groups, as those affected struggle to meet societal expectations of women and their caregiving roles [43]. 

Social identity theory facilitated this understanding of how various individuals perceive themselves or are perceived by others and how this shapes health-related experiences [44]. Individual social identities or positionalities provide the opportunity to examine health situations at a micro level, illustrating personal realities and particularities. Our identities situate us in space and give us a sense of belonging, influencing how we view ourselves and how others view us[18, 44]. Consequently, individuals possess diverse group memberships within societies; some are favoured, while others are oppressed, creating a clear social hierarchy, power dynamics, and differences in ways people experience their illness. These findings align with the existing body of literature regarding how social identities influence the experience of health issues. Evidence has shown that social identities relate to people’s daily experiences and affect how individuals manage health challenges and problems [19]. 

In contrast with research that has studied podoconiosis stigma with the focus on the experience of podoconiosis, types of stigma, and establishing podoconiosis as a stigmatising condition, comparing the experience of stigma between affected and non-affected[24], we documented inequality in the experience of podoconiosis stigma within affected groups due to individual identities. Affected individuals experience different amounts of podoconiosis stigma because of cultural constructs attached to their individual identities. Those with oppressive identities endure a higher burden of podoconiosis stigma than others, leading to inequality in the experience of stigma related to their illness. Within stigmatised groups because of podoconiosis, those with oppressive identities because of cultural constructs attached to them, are greatly stigmatised than others. These findings are consistent with other research on the differential experience of stigma due to individual identities [13, 45]. Also, our findings provide a comparison with studies that have explored risk factors of podoconiosis stigma, illuminating different levels of stigma across categories of persons affected with particular characteristics of differences such as income, gender, education, and employment [15]. This approach mainly utilises statistical calculations to measure the effects of these factors on the outcome, with little focus on individual identities and the cultural construction of identities. Our findings shed light on cultural nuances surrounding individual identities and how they shape inequalities in experiencing podoconiosis stigma. These findings complement previous research on the connection between individual identities and the experience of stigma related to illness, suggesting health disparities among persons affected [45–48]. 

Stigmatised individuals with oppressive identities experience multiplied instances of adversity, including lack of work opportunities, social exclusion, low self-esteem, and lack of access to essential services due to the fear of appearing in public, trapping those affected in a cycle of poverty and discrimination, contributing to health disparities, inequalities and poor well-being. These findings are congruent with the findings of a study conducted in Ethiopia that has documented discontinuation of seeking healthcare services, fearing the stigma [49, 50]. Similarly, our participants expressed concerns about appearing in public because of the stigma, particularly those with oppressive identities, which contribute to inadequate access to healthcare services. Those components of podoconiosis stigma, including denied work opportunities, poor access to basic services, and exclusion, have a significant effect on social, economic and mental health, and lead to health inequality, poverty and poor well-being of affected groups [8, 9, 12]. 

### Individual identities and coping strategies for podoconiosis stigma

Individual positioning due to their identities influences strategies for coping with podoconiosis stigma. People with marginalised identities struggled to adopt active strategies due to limited access to the necessary resources. The theory of coping links active coping strategies with positive impacts on psychosocial well-being [51]. Similarly, active coping strategies are linked with positive outcomes among people with podoconiosis in Ethiopia [10]. These include strategies involving resilience and treatment, such as regular hygiene or wearing shoes. In our study, strategies for hiding illness and "*Kwihambira*", which is a local term literally translates toperseverance included positive aspects of self-care and situational change. Wearing boots to hide swollen feet plays a dual purpose: prevention and management [52]. Hiding the affected parts improves social interactions and participation and contributes to feelings of safety and comfort. Although the literature on perseverance as a coping strategy is scant, our participants used this strategy, locally expressed, as a way of maintaining normal status, sustaining efforts to work and emotion regulation, showing resilience and going through their situation.

On the other hand, maintaining the ability to work to improve stigmatising identities is essential to perseverance strategies. However, individuals with marginalised identities, including being severely sick or poor, lacked the opportunity to adopt active strategies, trapping them in a vicious cycle. This lack of opportunities to choose could contribute to the psychological effects among those with minority identities, creating health disparities [51]. 

### Implications for policies and practices

Our findings shed light on the extreme health disparities and inequalities experienced by affected individuals with Stigmatising identities, suggesting the understanding of the podoconiosis stigma experience beyond the disease, considering individual identities related to gender, age, religion, and economic status in designing interventions to address health inequalities due to podoconiosis stigma. Inequality, poverty, and poor well-being among people affected by podoconiosis stigma are critical to eradicating poverty, reducing inequality, and addressing health disparities. This highlights the connection between podoconiosis-related stigma and national and global programs, including the pledge to leave no one behind and the SDGs. The experience of disparities and inequalities in experiencing podoconiosis-related stigma underscores the connection between disease-related stigma and SDGs, mainly 1, 8, and 10. SDG 1: End poverty in its all forms, mainly target 1.4: By 2030, ensure that all men and women, particularly the poor and the vulnerable, have equal rights to economic resources, as well as access to basic services, ownership and control over land and other forms of property, inheritance, natural resources, appropriate new technology and financial services, including microfinance; SDG 8: Decent work and economic growth – target 8.5: By 2030, achieve full and productive employment and decent work for all women and men, including young people and persons with disability, and equal pay work of equal value; and SDG 10: reduced inequalities – target 10.2 and 10.3: By 2030, empower and promote the social, economic, and political inclusion of all, irrespective of age, sex, disability, race, ethnicity, religion, economic or other status; and ensure equal opportunity and reduce inequalities of outcome, including by eliminating discriminatory laws, policies and practices and promoting appropriate legislation, policies and action in this regard, respectively [53]. The importance of addressing disease-related stigma in attaining the SDGs has increased [54]. Importantly, addressing inequalities due to disease-related stigma, considering the experience of stigma beyond podoconiosis as diseases, consider individual needs, culturally tailored approaches, and promoting positive individual identities may help implement people-centred approaches toward achieving the health equity and well-being of affected individuals.

### Strengths and limitations

The use of mixed qualitative methods and an extensive period of research fieldwork in the study area facilitated the richness of our findings, offering comprehensive insights into understanding of how individual identities contribute to inequalities in experiencing podoconiosis stigma, leading to health disparities. However, this study may have certain limitations. Issues of transferability may arise, as the findings presented in this paper are confined to a specific study area. However, the description of the study setting’s characteristics provides insight into the context, offering the transferability of the findings to contexts that share similar characteristics.

## Conclusion

Our study revealed disparities and inequalities in the experience of podoconiosis stigma among persons affected, which are shaped by individual identities. Within the stigmatised groups, some experience a greater amount of stigmatisation than others due to individuals’ identities, leading to extreme health disparities and inequalities among persons affected. Our findings offer valuable insights into understanding podoconiosis stigma beyond the disease, considering individual identities related to gender and economic status in specific contexts. This suggests the importance of integrating interventions to reduce podoconiosis-related stigma with other programs, such as education on gender equality and socio-economic empowerment services. This holistic approach can promote positive identities, self-esteem and social inclusion, contributing to the reduction of health inequities related to podoconiosis stigma, thus promoting the well-being of persons affected.

## Data Availability

The transcripts used and/or analysed for the current article are available in the Zenodo repository with the 10.5281/zenodo.17157683.
